# Efficacy and safety of hypofractionated radiotherapy for melanoma brain metastases: a retrospective study

**DOI:** 10.3389/fonc.2026.1782102

**Published:** 2026-04-02

**Authors:** Yacheng Wang, Heng Zhang, Zibo Tang, Dehua Kang, Weihao Xie, Xiaoshi Zhang, Lixia Lu

**Affiliations:** 1 Department of Oncology, The Central Hospital of Wuhan, Key Laboratory for Molecular Diagnosis of Hubei Province, Tongji Medical College, Huazhong University of Science and Technology, Wuhan, Hubei, China; 2 Department of Oncology, Longgang District Central Hospital of Shenzhen, Shenzhen, Guangdong, China; 3 Department of Radiation Oncology, Shenzhen People’s Hospital, The Second Clinical Medical College, Jinan University, The First Affiliated Hospital, Southern University of Science and Technology, Shenzhen, Guangdong, China; 4 Department of Radiation Oncology, Sun Yat-Sen University Cancer Center, Guangzhou, Guangdong, China

**Keywords:** brain metastases, hypofractionated radiotherapy, intracranial hemorrhage, local control, melanoma, safety

## Abstract

**Background and purpose:**

Melanoma brain metastasis (MBM) is associated with a poor prognosis and a high risk of intracranial hemorrhage (ICH), which may complicate the use of stereotactic radiosurgery (SRS). This study evaluated the efficacy and safety of hypofractionated radiotherapy (HFRT) for MBM, with a specific focus on metastases with hemorrhagic components.

**Patients and methods:**

In this single-center retrospective study, 26 patients with MBMs received HRT regimens of 30 Gy/5 fractions, 36 Gy/6 fractions, or 42 Gy/7 fractions. The primary endpoint was the local control (LC) rate assessed on the first MRI evaluation after radiotherapy completion (1–3 months after radiotherapy completion). Secondary endpoints included intracranial progression-free survival (IPFS), overall survival (OS), and treatment-related adverse events (AEs).

**Results:**

The disease control rate was 96% at the 3-month assessment (2 complete responses, 16 partial responses, 7 stable disease). The median OS was 10.5 months and the median IPFS was 4.5 months. No grade ≥3 AEs or symptomatic radiation necrosis occurred during the follow-up period (median 7.5 months). Notably, no radiotherapy-related ICH or exacerbation of pre-existing hemorrhage was observed, and hemorrhage reduction was achieved in all 11 patients with baseline ICH (100%).

**Conclusion:**

HFRT demonstrated promising local control and a favorable safety profile in this cohort of MBM patients, It shows particular potential for treating hemorrhagic metastases and may offer a valuable alternative to SRS in selected high-risk scenarios, meriting further investigation in larger studies.

## 1 Introduction

Melanoma is the third most common primary malignant tumor leading to brain metastases (accounting for 5% to 20%) (1). Clinical data indicate that up to 50% of patients with stage IV melanoma will develop brain metastases (MBMs) during the course of their disease, a figure that is even higher in autopsy results (exceeding 75%) (2). Brain metastases not only seriously affect patients’ quality of life but are also a leading cause of melanoma-related death. The median survival for patients with untreated brain metastases is reported to be only about 4 months (3). Furthermore, the time from the initial diagnosis of melanoma to the discovery of brain metastases ranges from less than 1 year to 5 years, with a median interval of 2.5 years (30.5 months) (4). For patients with a single, symptomatic brain metastasis, surgical resection of the intracranial lesion remains the gold standard for achieving local tumor control. However, ongoing advancements in radiotherapy have provided new treatment options for melanoma patients with brain metastases and improved clinical outcomes (5). With the advent of modern therapies including stereotactic radiosurgery (SRS) and effective systemic agents (immune checkpoint inhibitors and targeted therapies), survival outcomes have significantly improved, as evidenced by contemporary systematic reviews (6, 7).

Intracranial hemorrhage (ICH) is a serious clinical complication of brain metastases (BM) in melanoma patients, which occurs at a significantly higher rate than in BM from other primary tumor types (8). The high propensity for hemorrhage in MBM is attributed to their distinct pathological features, including unique vascularization patterns (e.g., vasculogenic mimicry and mosaic vessel formation), a dense vascular network, and increased vessel permeability (9). These characteristics, while supporting tumor growth, also significantly elevate the risk of spontaneous intratumoral hemorrhage (9). Intratumoral hemorrhage can accelerate disease progression and is a potentially life-threatening event. In patients with MBMs, stereotactic radiosurgery (SRS) is associated with a notable risk of new or increased hemorrhage, as reported in some studies, e.g., approximately 13.2% in a cohort analysis, with the majority of events within the first year (10). A comprehensive disease-specific systematic review confirmed that melanoma brain metastases carry a significantly elevated risk of post-SRS hemorrhage compared to brain metastases from other primary tumors (7). Additionally, SRS, which typically delivers high doses per fraction (e.g., 20–24 Gy for metastases <2 cm), is associated with a non-negligible risk of adverse radiation effects (AREs), particularly symptomatic radionecrosis (11). This risk is also a documented concern in melanoma patients, as confirmed by a melanoma-specific systematic review (7). Consequently, the presence of significant intratumoral hemorrhage is often considered a relative contraindication for SRS due to concerns about potentially increasing the risk of treatment-related complications (12). It should be noted that management is individualized, and alternative strategies such as a period of observation followed by SRS (including single-fraction or staged approaches) are valid options in selected cases. Whole-brain radiotherapy (WBRT) has been a conventional treatment approach. However, due to its large irradiation fields encompassing healthy brain tissue, WBRT can cause hippocampal injury and is associated with a significant risk of radiation-induced neurocognitive decline (13). In recent years, adjusted radiotherapy strategies, such as hypofractionated stereotactic radiotherapy, have been increasingly investigated to better balance local tumor control and safety, with the aim of reducing the incidence of serious AREs without compromising efficacy (14).

In this context, HFRT may represent an optimized strategy. This study aimed to describe the patient characteristics, treatment details, clinical outcomes, and adverse events in a cohort of patients with MBMs treated with HFRT, with a specific focus on its application in metastases with hemorrhagic components, in order to provide clinical data from a small sample on the efficacy and safety of HFRT in patients with melanoma brain metastases, and to offer a reference for optimizing clinical strategies.

## 2 Patients and methods

### 2.1 Patients

This retrospective study was approved by our Institutional Review Board, which granted a formal waiver of informed consent for the use and analysis of de-identified patient data. We collected data from 26 patients with malignant melanoma brain metastases treated at Sun Yat-sen University Cancer Center between January 2023 and October 2025. Patients meeting the following criteria were included: (1) histologically confirmed melanoma; (2) brain metastases confirmed by pre-treatment the magnetic resonance imaging (MRI), with no prior local therapy directed at these metastases; (3) Karnofsky performance status (KPS) score ≥70; (4) at least one measurable brain metastasis according to the Response Assessment in Neuro-Oncology Brain Metastases (RANO-BM) criteria; (5) at least one intracranial metastasis received a complete course of one of the HFRT regimens outlined in this study; (6) an MRI verification scan was performed before each fraction; (7) at least one post-radiotherapy follow-up imaging (1–3 months after radiotherapy completion) was available. Patients were excluded if they met any of the following criteria: (1) history of prior whole-brain radiotherapy or other radiotherapy for brain metastases; (2) surgical resection of brain metastases before radiotherapy; (3) lack of baseline imaging and follow-up imaging; (4) significant intratumoral hemorrhage before radiotherapy causing mass effect; (5) history of hypertension, known coagulation dysfunction, collagen vascular disease, or other preexisting conditions associated with intracranial hemorrhage; (6) diagnosis of another concurrent primary tumor; (7) incomplete clinical data.

### 2.2 Radiotherapy regimens

The decision to administer HFRT and the specific dose fractionation regimen were determined based on the patient’s condition or through multidisciplinary discussion, considering factors such as multiple metastases, larger lesion size, the presence of hemorrhagic components, or proximity to critical structures where single-fraction SRS might be associated with a higher perceived risk of complications.

All patients received HFRT. The specific regimens were as follows: The “6×5” regimen (30 Gy/5 fractions, 6 Gy per fraction, and a total dose of 30 Gy), the “6×6” regimen (36 Gy/6 fractions, 6 Gy per fraction, and a total dose of 36 Gy), and the “6×7” regimen (42 Gy/7 fractions, 6 Gy per fraction, and a total dose of 42 Gy). Treatments were delivered once daily, 5 days per week, and all regimens were completed within 2 weeks.

### 2.3 Data collection

The following data were collected for each enrolled patient: Baseline data: Demographic information (age, sex), primary site of melanoma, date of initial melanoma diagnosis, number of extracranial metastases, BRAF V600 mutation status. Brain metastasis characteristics: Number, maximum diameter, presence of intratumoral hemorrhage (determined by T1-weighted imaging (T1WI) and T2/susceptibility-weighted imaging (SWI) sequences on pre-radiotherapy MRI), radiotherapy regimen, lactate dehydrogenase (LDH) level, KPS score, systemic therapy administration: Use of systemic therapy during radiotherapy (defined as the period from 4 weeks prior to radiotherapy initiation until 4 weeks after its completion) (including immune checkpoint inhibitors of anti-PD-1/PD-L1 and anti-CTLA-4 agents, targeted therapy, and chemotherapy). The molecular graded prognostic assessment (molGPA) score was also calculated, as it has been demonstrated to be significantly associated with survival in melanoma patients.

### 2.4 Outcome measures and follow-up

The follow-up period was calculated from the date of initial diagnosis of intracranial metastases, using the pre-radiotherapy brain MRI as the baseline. Post-treatment follow-up brain MRI was performed at 1–3 months after radiotherapy completion, and subsequently every 3–6 months or as clinically indicated.

Primary efficacy endpoint: The local control (LC) rate was the primary endpoint. LC was defined as achieving a complete response (CR), partial response (PR), or stable disease (SD) according to the RANO-BM criteria at the first MRI evaluation after radiotherapy completion (1–3 months after radiotherapy completion): CR: Disappearance of all target lesions. PR: ≥30% decrease in the sum of the longest diameters of target lesions. SD: Changes not meeting the criteria for PR or progressive disease (PD). PD: ≥20% increase in the sum of the longest diameters of target lesions.

Secondary Efficacy Endpoints: Intracranial progression-free survival (IPFS): time from the start of radiotherapy to intracranial disease progression (any lesion) or death from any cause. Overall survival (OS): Time from the diagnosis of brain metastases to death from any cause or last follow-up.

Safety Endpoints: Hemorrhagic events: Defined as new hemorrhage in a brain metastasis or increased hemorrhage volume in a pre-existing metastatic lesion after radiotherapy. Events were assessed and graded based on imaging changes and clinical symptoms. Adverse Events(AEs): Included acute radiation-induced brain injury (e.g., headache, nausea/vomiting, fatigue, tinnitus, hearing impairment, speech difficulties) and symptomatic radiation necrosis (requiring confirmation by MRI perfusion-weighted imaging or pathology). All treatment-related toxicities were graded according to the Radiation Therapy Oncology Group (RTOG)/European Organisation for Research and Treatment of Cancer (EORTC) scoring system (15), which were assessed from the start of radiotherapy until the last follow-up.

### 2.5 Statistical analysis

Statistical analyses were performed using SPSS software, version 26.0. Categorical data are described using frequencies and percentages (%). Continuous data are described using the mean ± standard deviation or the median, as appropriate. Survival analysis was performed using the Kaplan-Meier method to calculate survival rates and plot survival curves, and group comparisons were made using the Log-rank test. For patients who had not experienced an endpoint event (such as death or disease progression) by the cutoff date, their survival time or progression-free survival time was treated as censored data. A two-sided P-value < 0.05 was considered statistically significant.

## 3 Results

### 3.1 Patient baseline characteristics

This study ultimately included 26 patients with melanoma brain metastases who received the defined HFRT regimens between January 2023 and October 2025. The median follow-up was 7.5 months (range: 1–28 months; mean 8.7 months). The baseline characteristics of the patients are detailed in **Table 1**. The median age was 53 years (range: 29-74). There were 12 males (46%) and 14 females (54%). All patients had a KPS score ≥70. The primary melanoma subtypes were cutaneous in 10 patients (38%), acral in 12 (46%), mucosal in 3 (12%), and other in 1 (4%). It should be specifically noted that among these, ‘acral’ specifically refers to the histopathological subtype of cutaneous melanoma known as acral lentiginous melanoma. A BRAF V600 mutation was detected in 12 patients (46.15%). The median interval from the initial melanoma diagnosis to the development of brain metastases was 27.5 months (range: 0–202 months; mean 43.03 months). At the time of brain metastasis diagnosis, elevated lactate dehydrogenase levels were observed in only 11 patients (42%). Twenty patients (77%) had received more than one line of systemic therapy prior to radiotherapy. According to the molGPA score, 8 patients (31%) had a score of 0.0–1.0, and 18 patients (69%) had a score of 1.5–4.0.

**Table 1:** Baseline patient characteristics.

Characteristics	Median (range) or *n* (%)
Patient number	(N = 26)
Age	53 (29-74)
Male	12 ( 46%)
Female	14 ( 54%)
KPS score	
70	3 ( 12%)
80	7 ( 27%)
90˜100	16 ( 62%)
Primary tumor type	
Cutaneous	10 ( 38%)
Acral (acral lentiginous)	12 ( 46%)
Mucosal	3 ( 12%)
Other	1 ( 4%)
BRAF V600 mutation	
Yes	12 ( 46%)
No	14 ( 54%)
LDH	
<ULN	15 ( 58%)
≥ULN	11 ( 42%)
Interval from initial diagnosis to BM, months	27.5 (0-202)
Lines of prior systemic therapy	
1	6 ( 23%)
>1	20 ( 77%)
Melanoma-molGPA	
0˜1.0	8 ( 31%)
1.5˜4.0	18 ( 69%)

BM, brain metastasis; KPS, Karnofsky Performance Status; LDH, lactate dehydrogenase; ULN, upper limit of normal; molGPA, Molecular graded prognostic assessment.

The ‘acral’ subtype specifically refers to acral lentiginous melanoma.

### 3.2 Brain metastasis characteristics and treatment

The median number of brain metastases was 4 (range: 1-11). The median maximum diameter of brain metastases was 18 mm (range: 4–38 mm). Extracranial metastases were present in all 26 patients (100%); 24 patients (92%) had more than 3 extracranial metastatic sites. At the initial diagnosis of brain metastases, 11 patients (42%) had brain metastases with associated hemorrhage.

Regarding the radiotherapy regimen, 2 patients (8%) received the “6x5” regimen, 19 patients (73%) received the “6x6” regimen, and 5 patients (19%) received the “6x7” regimen. 20 patients (77%) received at least one form of systemic treatment during radiotherapy, while 6 (23%) did not. Among those treated, 14 patients (54%) received immunotherapy, 12 patients (46%) received targeted therapy, and 14 patients (54%) received chemotherapy. The details of the concurrent systemic therapy regimens for each patient are provided in **Supplementary Table 1**.

The characteristics of the brain metastases and treatments are detailed in **Table 2**.

**Table 2:** Baseline brain metastasis characteristics and treatment.

Characteristics	Median (range) or *n* (%)
Total number of BM	
1	8 ( 31%)
2˜4	9 ( 35%)
5˜10	8 ( 31%)
>10	1 ( 4%)
Maximum diameter of BMs, mm	18 mm (4mm-38mm)
BMs with hemorrhage	11 ( 42%)
Radiotherapy regimen	
6×5 (30 Gy/5 fx)	2 ( 8%)
6×6 (36 Gy/5 fx)	19 ( 73%)
6×7 (42 Gy/5 fx)	5 ( 19%)
Number of extracranial metastatic sites	
0	0 (0%)
1˜3	2 ( 8%)
≥ 4	24 ( 92%)
Systemic therapy concurrent with radiotherapy	
Immunotherapy	14 ( 54%)
Targeted therapy	12 ( 46%)
Chemotherapy	14 ( 54%)

BM, Brain Metastasis; fx, fractions.

Percentages are based on the total number of patients (N = 26).

### 3.3 Treatment efficacy

**Figure 1:**
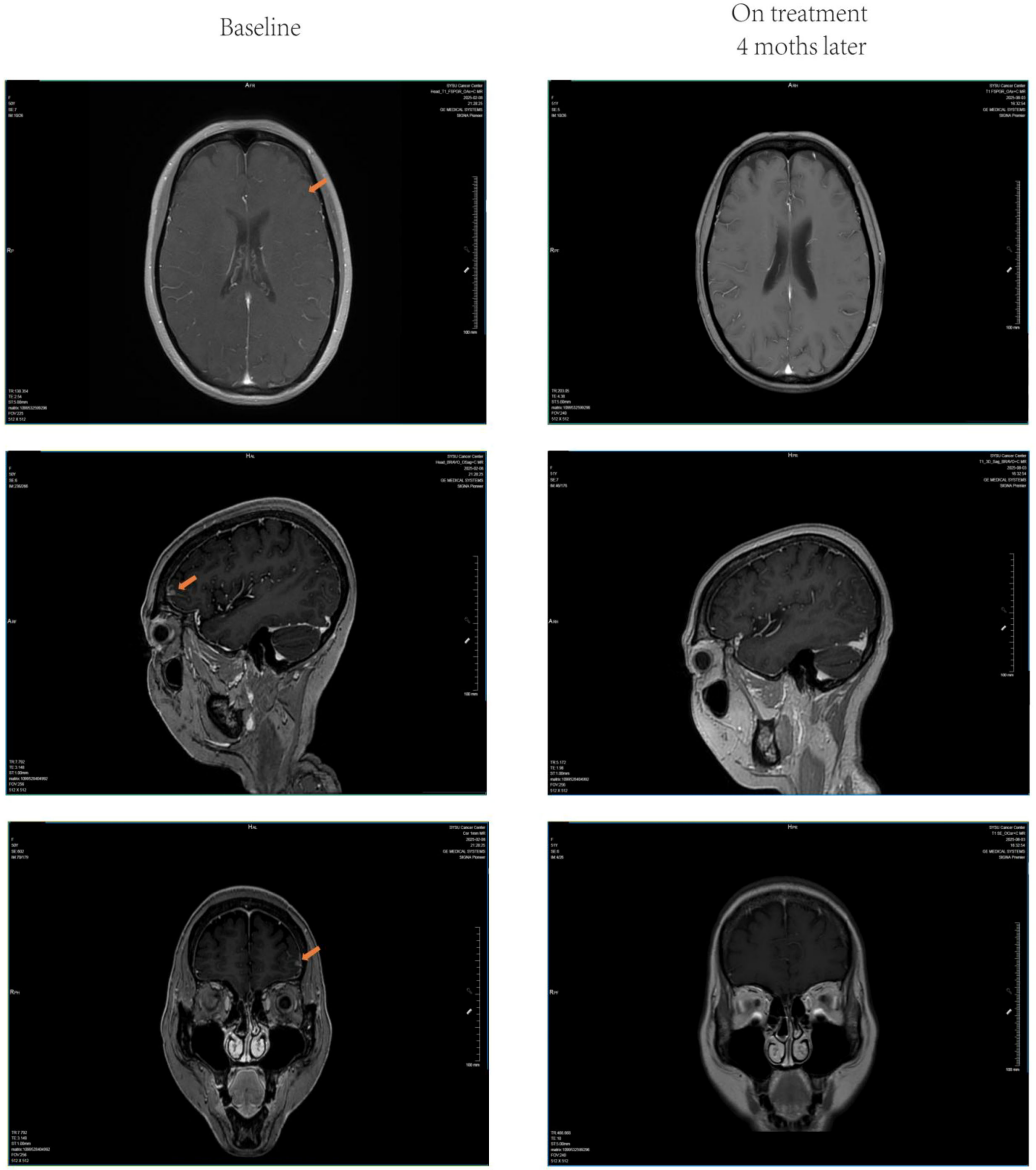
Imaging example of intracranial complete response. A 51-year-old female with melanoma brain metastases treated with a hypofractionated radiotherapy regimen of 6 Gy per fraction for 6 fractions. The magnetic resonance imaging (MRI) shows complete response of the intracranial metastatic lesion at 4-month follow-up. (The orange arrow indicates the lesion).

**Figure 2:**
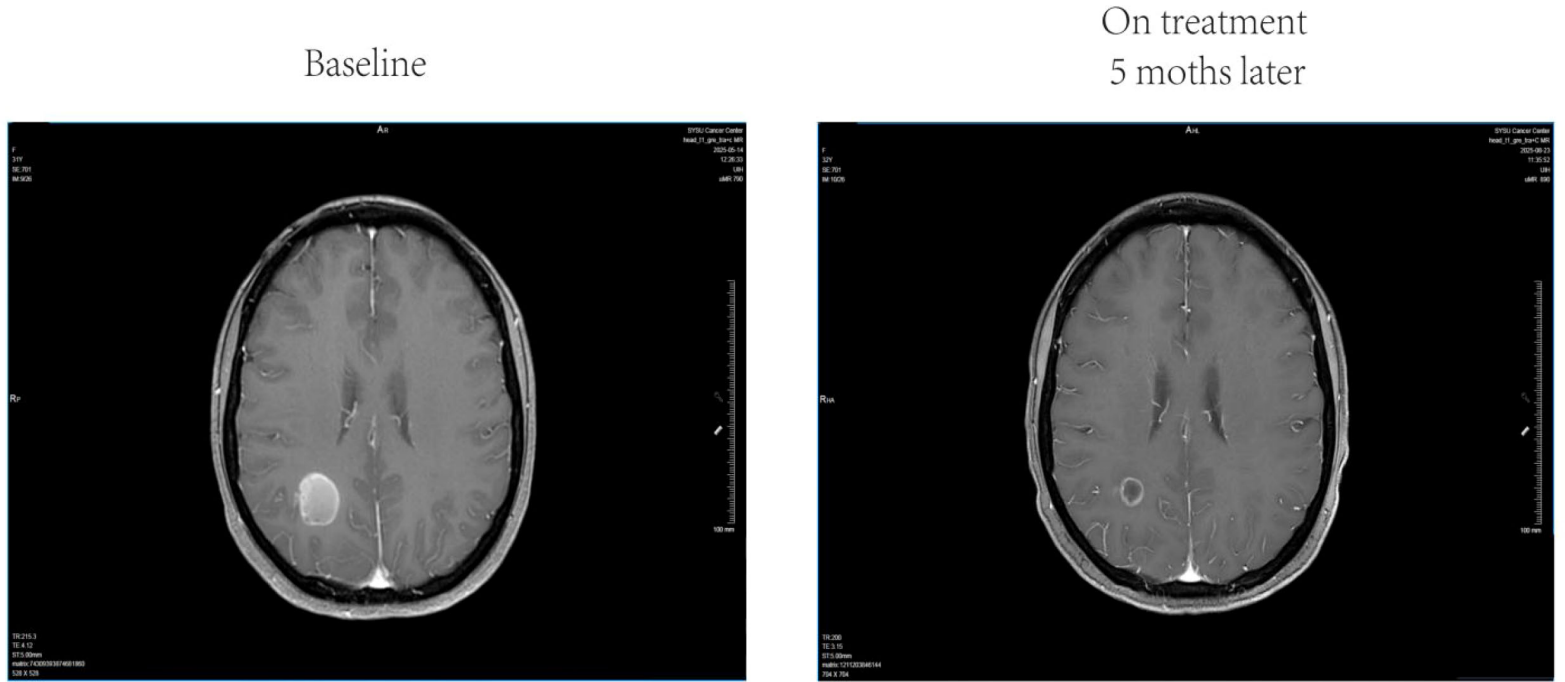
Imaging case of intracranial partial response. A 32-year-old female with melanoma brain metastases treated with a hypofractionated radiotherapy regimen of 6 Gy × 6 fractions. The magnetic resonance imaging (MRI) shows partial response at 5-month follow-up.

Disease LC after radiotherapy was observed in 25 patients (96%). This included 2 patients (8%) with a CR, 16 patients (62%) with a PR, and 7 patients (27%) with SD. One patient (4%) experienced intracranial progression. Descriptively, the local control rate was 84% for metastases <2 cm and 78% for metastases ≥2 cm. **Figure 1** presents the MRI of a patient who achieved a CR, and **Figure 2** depicts the MRI of a patient with a PR. Throughout the follow-up period, intracranial progression occurred in 15 patients (58%), 5 of whom patients developed new intracranial lesions.

**Figure 3:**
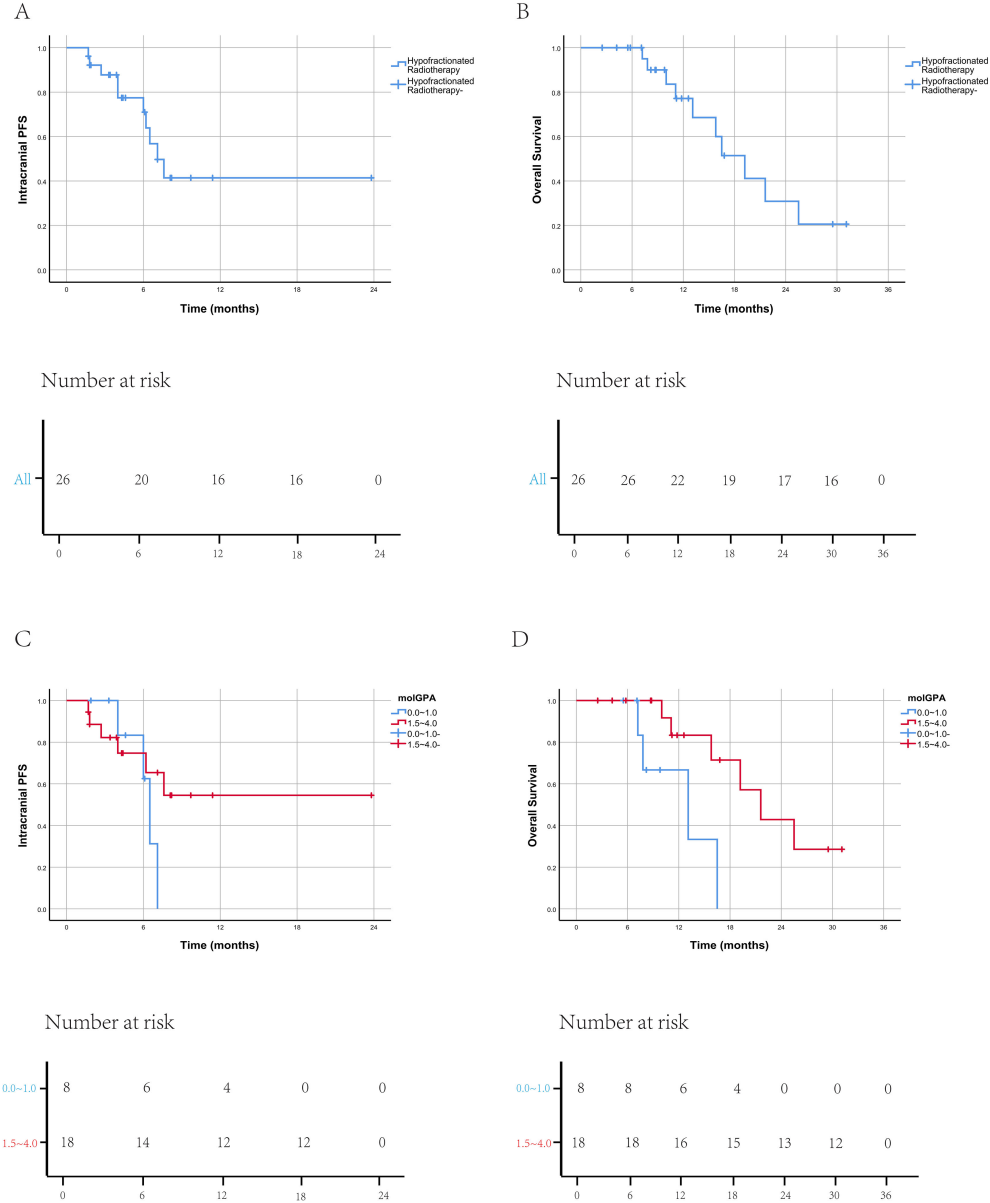
Analysis of overall survival and intracranial progression-free survival. **(A, B)** Overall survival (OS) **(A)** and intracranial progression-free survival (IPFS) **(B)** of melanoma brain metastasis patients treated with hypofractionated radiotherapy. **(C, D)** Comparison of OS **(C)** and IPFS **(D)** between patients with molGPA scores of 0.0-1.0 and those with scores of 1.5-4.0.

The median OS for patients in this study was 10.5 months (range: 2.4–30.7 months) (**Figure 3B**), and the median IPFS was 4.5 months (range: 1.7–23.7 months) (**Figure 3A**). At the 1-year follow-up, 22 patients were still alive, and the 1-year OS rate was 85%. A total of 9 patients (35%) were deceased. The deceased patients died between 7.2 and 21.6 months from brain metastasis diagnosis. Patients with low molGPA scores (0.0-1.0) had a lower OS compared to those with high scores (1.5-4.0), and there is a statistically significant difference (P < 0.05) (**Figure 3C**). However, IPFS did not show a statistically significant difference between the two score groups (P > 0.05) (**Figure 3D**).

### 3.4 Treatment-related adverse events

**Figure 4:**
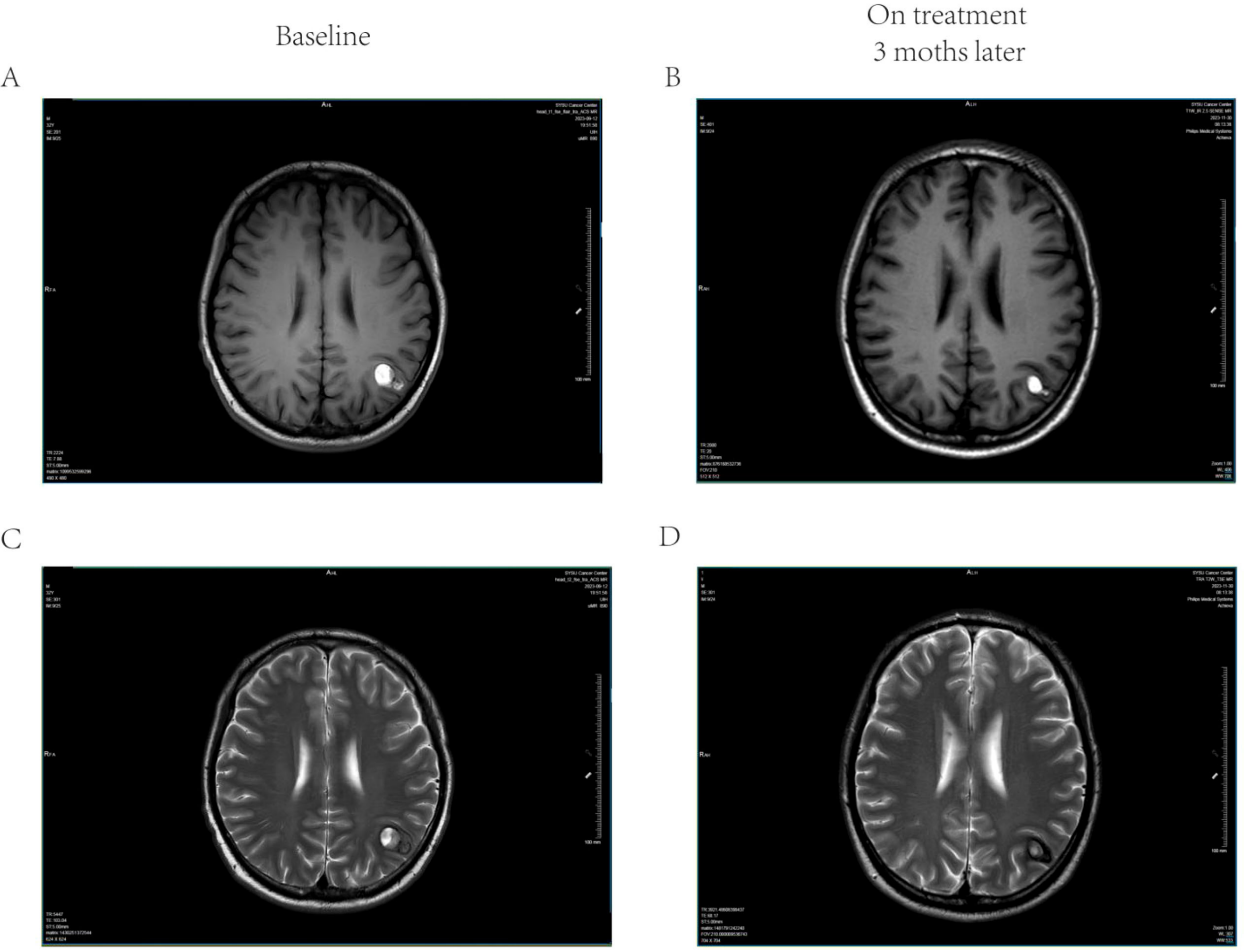
Representative neuroimaging of a hemorrhagic melanoma brain metastasis before and after hypofractionated radiotherapy. Axial magnetic resonance imaging (MRI) sections of a 32-year-old male patient with a hemorrhagic melanoma brain metastasis treated with a hypofractionated stereotactic radiotherapy regimen (6 Gy × 6 fractions). **(A)** T1-weighted imaging(T1W1) at baseline shows a lesion with intrinsic T1 shortening, suggestive of hemorrhage or melanin content. **(B)** Corresponding T2-weighted or susceptibility-weighted imaging **(SWI)** sequence at baseline confirms significant magnetic susceptibility, indicative of intratumoral hemorrhage. **(C)** Follow-up T1-weighted image obtained 3 months after radiotherapy demonstrates a significant reduction in lesion size and T1 hyperintensity. **(D)** Follow-up T2/SWI sequence obtained 3 months after radiotherapy confirms marked reduction in hemorrhagic components. The imaging findings illustrate the reduction in both tumor volume and associated hemorrhage following treatment.

The incidence of treatment-related toxicities is summarized in **Table 3**. The median follow-up period was 7.5 months. No grade 3 or higher adverse events occurred. Headache was the most frequently reported toxicity (n=8, 31%). No grade 3 or higher acute radiation-induced brain injury was observed. During the follow-up period, no cases of symptomatic radiation necrosis were identified. Crucially, no radiotherapy-related symptomatic intracranial hemorrhage or exacerbation of pre-existing hemorrhage occurred. Notably, hemorrhage reduction was achieved in all 11 patients who had baseline intracranial hemorrhage (100%). Representative MRI images of a melanoma brain metastasis patient with hemorrhage at baseline and 3 months after radiotherapy are shown in **Figure 4**. Overall, no patient had discontinued treatment due to adverse events.

**Table 3:** Radiation toxicity (RTOG/EORTC).

Symptom and grade	Grade 1	Grade 2	Grade 3-5	All (%)
Headache	5	3	0	8 (31%)
Nausea/Vomiting	1	0	0	1 (4%)
Fatigue	4	1	0	5 (19%)
Tinnitus	1	0	0	1 (4%)
Hearing Impairment	1	0	0	1 (4%)
Speech Disorder	1	0	0	1 (4%)

RTOG, Radiation Therapy Oncology Group; EORTC, European Organisation for Research and Treatment of Cancer.

## 4 Discussion

Melanoma is recognized as one of the malignancies with the highest propensity for brain metastasis (16). Recent studies indicate that the cumulative incidence of brain metastases in melanoma patients can be as high as 37% over the course of the disease (17). Analyses of modern patient cohorts underscore a significant improvement in survival outcomes attributable to contemporary multimodal therapies. For instance, one study focusing on patients treated between 2015 and 2021 reported a median overall survival (OS) of 16.6 months from the diagnosis of brain metastases (18). Corroborating this positive trend, real-world evidence confirms a substantial improvement in OS for patients diagnosed after 2015, with median OS figures ranging from approximately 6.9 to 13.0 months, a benefit largely attributed to the adoption of immune checkpoint inhibitors and targeted therapies (18–20). While the prognosis for melanoma patients with MBMs remains challenging, these data collectively highlight the impact of modern systemic therapies. The challenge of delivering therapeutic agents across the blood-brain barrier limits the efficacy of chemotherapy in improving OS for MBM patients (21). While targeted therapies (e.g., BRAF/MEK inhibitors) and immunotherapy (e.g., immune checkpoint inhibitors) have markedly improved outcomes for melanoma brain metastases (22), radiotherapy continues to be a critical treatment modality. Local treatments, including surgery and SRS, with or without systemic therapy, can prolong OS in these patients (23). Although SRS has proven more effective than WBRT in extending OS while preserving neurocognitive function (24–27), both modalities are associated with significant side effects, notably neurocognitive decline with WBRT and radiation necrosis with SRS (28). For larger or complex lesions, fractionated regimens like HFRT or adaptive volume-staged SRS are increasingly used to improve the therapeutic ratio (29, 33). Unlike single-fraction SRS which delivers a high dose in one session, this hypofractionated approach administers a moderate dose per fraction over multiple sessions, aiming to maintain efficacy while potentially reducing the risk of adverse radiation effects. Melanoma has traditionally been considered relatively radioresistant and is associated with a high risk of hemorrhage (29). For patients with larger or complexly located metastases, fractionated radiotherapy regimens, which employ lower doses per fraction, may achieve a better balance between local control and toxicity, potentially reducing acute vascular damage and treatment-related hemorrhage risk (29, 30).However, data on reduced-dose fractionated radiotherapy for MBM remain scarce, underscoring the need for alternative fractionated regimens with optimal efficacy and safety (29, 30).

In this study, a HFRT regimen resulted in disease control in 25 patients (96%), comprising 2 (8%) with complete response and 16 (62%) with partial response, indicating a favorable local tumor control rate that appears superior to rates reported in some studies of hypofractionated stereotactic radiotherapy or SRS for MBM (29). Regarding AREs, standard-dose SRS has been associated with a higher incidence of AREs compared to reduced-dose regimens, particularly with concurrent BRAF inhibitor use (31, 32). Our findings suggest that the lower fractional doses used in our regimen may reduce the risk of AREs compared to higher-dose SRS. The absence of grade 3 or higher acute treatment-related AEs and the fact that no patient discontinued treatment due to AEs indicate good tolerability. Hypofractionation offers radiobiological advantages over single-fraction SRS by allowing normal tissue repair between fractions, potentially enabling a higher biologically effective dose while reducing the risk of radiation necrosis, which is a significant concern for larger metastases or those near critical structures where delivering a curative dose with SRS is challenging (33–35). This theoretical advantage is supported by recent analyses showing comparable or superior local control with hypofractionation for larger brain metastases alongside a lower risk of symptomatic radiation necrosis (36). The absence of symptomatic radiation necrosis in our cohort during follow-up is consistent with this, though longer observation is needed. Importantly, for hemorrhagic MBM—often approached with caution for single-fraction SRS due to reports of new or increased hemorrhage post-SRS (37–39)—our regimen provided a viable local control option. Fractionated radiotherapy may mitigate hemorrhage risk through gradual vascular remodeling (11). Notably, we observed no new symptomatic ICHs or exacerbation of pre-existing hemorrhages related to treatment, and hemorrhage reduction was achieved in all patients with baseline hemorrhagic metastases, demonstrating promising efficacy and safety for MBM patients with hemorrhage.

Our findings evaluated a HFRT regimen in patients with MBMs. The cohort demonstrated a median OS of 10.5 months and a median IPFS of 4.5 months. These outcomes compare favorably with historical data from MD Anderson Cancer Center, which reported median OS of 9.8 months for surgery, 7.7 months for SRS, 4.6 months for chemotherapy, and 3.9 months for WBRT. The prolonged survival observed suggests that this hypofractionated regimen is an effective and well-tolerated treatment option for MBM, offering a promising balance between efficacy and safety (40). Precise prognostic stratification is crucial for individualized treatment planning. The molGPA score, which incorporates KPS, number of brain metastases, extracranial disease status, serum LDH, and BRAF mutation status, helps identify patients most likely to benefit from aggressive local intervention (41). For instance, patients with high molGPA scores may achieve median survival of several years, warranting aggressive local strategies for long-term intracranial control (42). Our results, showing significantly lower OS in patients with low molGPA scores compared to those with high scores, are consistent with the literature and confirm the prognostic value of molGPA for OS in MBM patients (43). Future studies could more systematically use molGPA to optimize patient selection for HFRT. The lack of a significant correlation between molGPA score and IPFS in our study might be explained by the relatively consistent initial local control achieved with modern hypofractionated techniques across different prognostic groups, potentially flattening differences in IPFS (44). Conversely, the molGPA score’s stronger correlation with OS likely reflects its ability to assess systemic tumor burden, with extracranial disease progression being a major determinant of ultimate survival outcome, an aspect not captured by IPFS (43, 45). Furthermore, in the era of effective systemic therapies, the impact of these treatments on survival may further accentuate the predictive role of molGPA for OS while limiting its value for predicting purely intracranial progression (45).

This study has limitations. Its retrospective, single-center design and the relatively small sample size inherent to the disease incidence may introduce selection bias. The small, heterogeneous cohort (in terms of disease burden, BRAF status, metastasis size, and systemic therapies etc.) limits the generalizability of findings and precludes definitive subgroup analyses. Although survival data were meticulously recorded, their collection was not prospective or standardized. Standardized patient-reported symptom assessments were not available, limiting our evaluation of symptomatic relief. Therefore, future multicenter, large-scale prospective trials are needed to validate the application of this HFRT regimen in MBM patients. Secondly, patient heterogeneity and variations in systemic treatments received could have influenced the outcomes but reflected real-world practice. With combination immunotherapy becoming the first-line standard for asymptomatic MBM, significantly improving patient survival, and with the timing of immunotherapy relative to radiotherapy being crucial for potential synergistic effects (46, 47), the observed trend toward better efficacy in patients receiving combined immunotherapy in our study warrants further investigation in larger cohorts to delineate the synergistic effects and optimal sequencing of this radiotherapy regimen with immunotherapy.

In summary, this retrospective analysis of 26 MBM patients treated with HFRT, particularly focusing on lesions with hemorrhage risk, demonstrated promising local control and a favorable safety profile. The proposed regimen appears to be an optimized strategy balancing efficacy and AREs for MBM, showing particular potential for patients with hemorrhagic brain metastases, who often face limited options due to traditional radiotherapy contraindications.

## Data Availability

The original contributions presented in the study are included in the article/**Supplementary Material**. Further inquiries can be directed to the corresponding author.
