# Supplementary material for: Efficacy and safety of hypofractionated radiotherapy for melanoma brain metastases: a retrospective study
Source: Front Oncol. 2026 Apr 2;16:1782102. doi: 10.3389/fonc.2026.1782102 (PMC13082948; doi:10.3389/fonc.2026.1782102)
Supplement: Supplementary file 1 [file Table1.docx]

Supplementary Material

# Supplementary Table

**Table S1. Systemic Therapies of Enrolled Patients**

| **Patient Number** | **Systemic Therapy Received During Radiotherapy** |
| --- | --- |
| 1 | albumin-paclitaxel + pembrolizumab + ipilimumab |
| 2 | dabrafenib + trametinib |
| 3 | No concurrent systemic therapy |
| 4 | pembrolizumab + ipilimumab |
| 5 | dabrafenib + trametinib + temozolomide |
| 6 | albumin-paclitaxel + bevacizumab + pembrolizumab |
| 7 | albumin-paclitaxel + toripalimab + bevacizumab |
| 8 | temozolomide + pembrolizumab |
| 9 | No concurrent systemic therapy |
| 10 | dabrafenib + trametinib + toripalimab |
| 11 | pembrolizumab |
| 12 | lenvatinib + paclitaxel + carboplatin + toripalimab |
| 13 | temozolomide + pembrolizumab + axitinib |
| 14 | pembrolizumab + bevacizumab |
| 15 | No concurrent systemic therapy |
| 16 | temozolomide |
| 17 | No concurrent systemic therapy |
| 18 | temozolomide + pembrolizumab |
| 19 | temozolomide + pembrolizumab+ axitinib |
| 20 | No concurrent systemic therapy |
| 21 | temozolomide + axitinib + toripalimab |
| 22 | bevacizumab |
| 23 | nivolumab + ipilimumab + temozolomide |
| 24 | albumin-paclitaxel + carboplatin |
| 25 | No concurrent systemic therapy |
| 26 | dabrafenib + trametinib + temozolomide |
